# Expression of a Human Caveolin-1 Mutation in Mice Drives Inflammatory and Metabolic Defect-Associated Pulmonary Arterial Hypertension

**DOI:** 10.3389/fmed.2020.00540

**Published:** 2020-09-11

**Authors:** Anandharajan Rathinasabapathy, Courtney Copeland, Amber Crabtree, Erica J. Carrier, Christy Moore, Sheila Shay, Santhi Gladson, Eric D. Austin, Anne K. Kenworthy, James E. Loyd, Anna R. Hemnes, James D. West

**Affiliations:** ^1^Allergy, Pulmonary and Critical Care Medicine, Vanderbilt University Medical Center, Nashville, TN, United States; ^2^Pulmonary and Critical Care Medicine, Brigham and Women's Hospital and Harvard Medical School, Boston, MA, United States; ^3^Pediatrics, Vanderbilt University Medical Center, Nashville, TN, United States; ^4^Molecular Physiology and Biophysics, Vanderbilt University School of Medicine, Nashville, TN, United States

**Keywords:** Cav1, pulmonary hypertension, exercise, inflammation, monocyte, metabolism

## Abstract

**Background:** In 2012, mutations in Cav1 were found to be the driving mutation in several cases of heritable pulmonary arterial hypertension (PAH). These mutations replaced the last 21 amino acids of Cav1 with a novel 22-amino-acid sequence. Because previously only Cav1 knockouts had been studied in the context of PAH, examining the *in vivo* effects of this novel mutation holds promise for new understanding of the role of Cav1 in disease etiology.

**Methods:** The new 22 amino acids created by the human mutation were knocked into the native mouse Cav1 locus. The mice underwent hemodynamic, energy balance, and inflammatory measurements, both at baseline and after being stressed with either a metabolic or an inflammatory challenge [low-dose lipopolysaccharide (LPS)]. To metabolically challenge the mice, they were injected with streptozotocin (STZ) and fed a high-fat diet for 12 weeks.

**Results:** Very little mutant protein was found *in vivo* (roughly 2% of wild-type by mass spectrometry), probably because of degradation after failure to traffic from the endoplasmic reticulum. The homozygous mutants developed a mild, low-penetrance PAH similar to that described previously in knockouts, and neither baseline nor metabolic nor inflammatory stress resulted in pressures above normal in heterozygous animals. The homozygous mutants had increased lean mass and worsened oral glucose tolerance, as previously described in knockouts. Novel findings include the preservation of Cav2 and accessory proteins in the liver and the kidney, while they are lost with homozygous Cav1 mutation in the lungs. We also found that the homozygous mutants had a significantly lower tolerance to voluntary spontaneous exercise than the wild-type mice, with the heterozygous mice at an intermediate level. The mutants also had higher circulating monocytes, with both heterozygous and homozygous animals having higher pulmonary MCP1 and MCP5 proteins. The heterozygous animals also lost weight at an LPS challenge level at which the wild-type mice continued to gain weight.

**Conclusions:** The Cav1 mutation identified in human patients in 2012 is molecularly similar to a knockout of Cav1. It results in not only metabolic deficiencies and mild pulmonary hypertension, as expected, but also an inflammatory phenotype and reduced spontaneous exercise.

## Introduction

Caveolae are flask-shaped invaginations in the plasma membrane, typically about 50–100 nm in diameter. They are abundant in endothelial cells, smooth muscle, and adipocytes but also present in many other cell types (1). They serve many cellular functions, including buffering mechanical stress, maintaining membrane integrity, and regulating endocytosis (2). They are also involved in many signal transduction pathways, although their exact role is still under investigation.

Caveolin-1 (Cav1) is an essential component of caveolae in most tissues. It is a 178-amino-acid integral membrane protein that inserts into the membrane in a hairpin structure, generally as part of a complex of 14–16 caveolin monomers (generally incorporating both caveolin 1 and 2). The caveolae assembly also involves several accessory proteins, including cavins, PACSIN2, and EHD-2 (3).

Until 2012, the disease with the clearest association with Cav1 mutations had been lipodystrophy (4), although associations between caveolae function and disease had been drawn for multiple other conditions, including cancer, muscular dystrophy, and cardiovascular disease (5, 6). There was an extensive literature on Cav1 knockout driving pulmonary hypertension in mice (7–10), but this was largely theoretical since it had not been seen in humans. Overall, though, Cav1 knockout mice are viable and fertile, and their health problems are unobvious until in advanced age.

However, in 2012, Cav1 mutations were found as the likely causative mutations in a familial pulmonary hypertension family (11), and since then, causative mutations have been found in additional families, although it remains a rare cause. Other examples include a F160X Cav1 frame shift mutation associated with both pulmonary arterial hypertension (PAH) and congenital generalized lipodystrophy that causes premature truncation of the protein (12–14). In addition, in a recent whole-exome sequencing of 2,572 cases, potentially causative CAV1 mutations were found in 10 individuals, representing seven families or isolated cases (15).

The mutation identified in that first family was a frameshift mutation near the end of the protein P158P fsX22. This replaced the final 21 amino acids, which were known to make up a trafficking signal (16), with an entirely novel 22 amino acids. That this resulted in pulmonary hypertension in humans as heterozygous was interesting since, in mice, only homozygous knockouts developed the disease. This implied the possibility of a dominant negative effect—since caveolins assemble into oligomers, one might imagine that the mutant proteins within these structures either retain the wild-type protein in the endoplasmic reticulum (ER) or Golgi or are trafficked to the plasma membrane and form caveolae with the wild-type, resulting in some dysfunction of the caveolae. We thus set to work in building a mouse with a knock-in of the human mutation into the native mouse Cav1 locus shortly after its initial publication in 2012.

In the interim, both we and others took a closer look at how the mutation worked in cell culture models. On its own, the P158P fsX22 mutation was trapped in the endoplasmic reticulum; however, when co-expressed with wild-type Cav1, it appeared to act as a partial dominant negative, with fewer caveolae and with compromised function as membrane reservoir, in addition to potential signaling defects (17, 18). The goal of the present study was to determine whether these defects were recapitulated *in vivo* in mice and their physiological consequences.

## Materials and Methods

### Reagents and Chemicals

Streptozotocin (STZ), lipopolysaccharide (LPS), and all the other fine chemicals were purchased from Sigma Aldrich (St. Louis, MO, USA). High-fat diet (HFD) was purchased from Bio-Serv (Flemington, NJ, USA). Caveolin (610060) and Caveolin 2 (610684) antibodies were purchased from BD Biosciences (San Jose, CA, USA). Cavin1 (ab48824), EHD2 (ab23935), β-actin (ab8227), and CD68 (ab125212) antibodies were obtained from Abcam (Cambridge, MA, USA), α-smooth muscle actin was from Dako (Santa Clara, CA, USA), and PACSIN2 (AP8088b) antibody was from Abcepta (San Diego, CA, USA). Proteome Profiler Mouse Cytokine Array Kit was purchased from R&D systems (Minneapolis, MN, USA).

### Animals

All animal studies were reviewed and approved by the Institutional Animal Care and Use Committee at the Vanderbilt University Medical Center (approval numbers M/12/106 and M/11/207), in compliance with the National Institutes of Health guidelines. All these studies are conducted in accordance with the ARRIVE guidelines for reporting experiments involving animals (19).

### Generation of Transgenic Mice Expressing Human CAV1 Mutation

Heterozygous (Cav1^+/P^) and homozygous (Cav1^P/P^) animals expressing the frame shift human caveolin 1 mutation (P158P fsX22) were generated on a mixed C57/Bl6J and FVB/N background. This was accomplished with a traditional knock-in into embryonic stem cells, with the mutation inserted into the native locus of the mouse CAV1 exon 3 and with the recombination arms extending into intron 2 and the 3′ genomic sequence. Selection markers were included in intron 2 but excised during the final creation of the mouse.

### Study Design

Two different animal studies were performed apart from a pilot phenotyping investigation. In the phenotyping study, we had two sets of groups. Nine- to 11-weeks-old (Cav1^+/+^, *n* = 4; Cav1^+/P^, *n* = 4; Cav1^P/P^, *n* = 2) and 28- to 31-weeks-old (Cav1^+/+^, *n* = 8; Cav1^+/P^, *n* = 15) animals in the first (younger) and second (older) groups were subjected to hemodynamic assessments. In the subsequent studies, the animals were either exposed to high-fat diet + streptozotocin (diet study) or lipopolysaccharide (LPS study). Unless specified, all animals throughout the studies were exposed to 12:12-h dark/light cycle with unlimited access to regular chow and water. Both sexes (male and female) were used in all studies.

- Diet study
  In this study, 25- to 58-weeks-old animals were fed either on regular chow (13.5% fat) or 60% high-fat, high-calorie diet for 4 months. This study included six groups in two subsets. Each subset had three groups each of wild-type, heterozygous, and homozygous Cav1 animals—one set on regular chow and the other on HFD. In the first subset (HFD), after 4 weeks of HFD exposure, the animals were fasted for 4 h and streptozotocin (STZ) was administered (150 mg/kg, i.p. route). Following 3 days of STZ administration, blood glucose was estimated to confirm diabetes (blood glucose, >250 mg/dl). A second dose (half-dose) of STZ was administered (on the 5th week) to the animals, which did not develop diabetes following a single dose of STZ. Subsequent to STZ administration, the animals were continued on HFD for another 8 weeks. Blood glucose was monitored in all animals every 4 weeks—week 0 and end of weeks 4, 8, and 12. After a week of acclimatization in individual metabolic cages (week 10), all animals underwent an extensive indirect calorimetry energy expenditure assessment for a continuous 72 h in the Vanderbilt Mouse Metabolic Phenotypic Center. On the 11th week, an oral glucose tolerance test (OGTT) was performed on all animals, followed by 4 h of fasting. Following OGTT, the animals were continued on HFD until the experiments were terminated. Transthoracic echocardiography was performed on all animals before the hemodynamic assessment. This HFD subset included three groups, Cav1^+/+^ (*n* = 13), Cav1^+/P^ (*n* = 15), and Cav1^P/P^ (*n* = 16). All animals, Cav1^+/+^ (*n* = 10), Cav1^+/P^ (*n* = 8), and Cav1^P/P^ (*n* = 20), in the next subset were exposed to regular chow throughout the study and subjected to the same experimental parameters, as mentioned previously.
- LPS study
  Similar to the diet study, we had six groups of animals spread in this study between two subsets, aged between 42 and 76 weeks. In the first subset (LPS), the animals fed on high-energy chow (25.4% fat) were challenged with LPS (7.5 μg/dose/intratracheal route) every 4 days, for a cumulative dose of 45 μg over 3 weeks. All the animals were provided with saline and supplemental gel food and eventually HFD, if they were sick. This subset included the following groups: Cav1^+/+^ (*n* = 3), Cav1^+/P^ (*n* = 10), and Cav1^P/P^ (*n* = 4). After 21 days of LPS exposure, the study was terminated following a hemodynamic assessment. In the second subset (vehicle), all the experimental parameters as explained above were adopted, except LPS, which was replaced with the vehicle (PBS). This subset had three groups: Cav1^+/+^ (*n* = 3), Cav1^+/P^ (*n* = 3), and Cav1^P/P^ (*n* = 2).

### Energy Expenditure Assessment by Indirect Calorimetry Method

For the energy expenditure assessment, the animals were individually placed in metabolic cages (identical to home cages with bedding) in a 12-h light/dark cycle, temperature/humidity-controlled dedicated room in the Vanderbilt Mouse Metabolic Phenotyping Core. Energy expenditure measures were obtained by indirect calorimetry (Promethion, Sable Systems, Las Vegas, NV, USA). The calorimetry system consisted of cages identical to home cages, with the bedding equipped with water bottles and food hoppers connected to load cells for food and water intake monitoring. All animals had *ad-libitum* access to either regular diet or HFD and water. The air within the cages was sampled through microperforated stainless steel sampling tubes that ensure uniform cage air sampling. Promethion utilizes a pull-mode, negative pressure system with an excurrent flow rate set at 2,000 ml/min. Water vapor was continuously measured, and its dilution effect on O_2_ and CO_2_ was mathematically compensated for the analysis stream (20). O_2_ consumption and CO_2_ production were measured for each mouse every 5 min for 30 s. Incurrent air reference values were determined every four cages. The respiratory quotient was calculated as the ratio of CO_2_ production over O_2_ consumption. Energy expenditure was calculated using the Weir equation: EE (kcal/h) = 60 ^*^ [0.003941 ^*^ VO_2_ (ml/min) + 0.001106 ^*^ VCO_2_ (ml/min)] (21). Ambulatory activity was determined every second with XYZ beams. Data acquisition and instrument control were coordinated by MetaScreen v2.2.18, and the raw data were processed using ExpeData v1.7.30 (Sable Systems). Finally, the fat and the lean masses were estimated by NMR on the Bruker Minispec (Bruker Biospin Corporation, Bellerica, MA, USA). This study included the following randomly chosen animals: (i) regular chow subset—Cav1^+/+^ (*n* = 10), Cav1^+/P^ (*n* = 8), and Cav1^P/P^ (*n* = 8) and (ii) HFD subset—Cav1^+/+^ (*n* = 9), Cav1^+/P^ (*n* = 10), and Cav1^P/P^ (*n* = 8).

### Oral Glucose Tolerance Test

On the 11th week of the study, oral glucose tolerance test (OGTT) was performed on the same animals discussed in the energy balance study. For OGTT, all animals fasted for 4 h were administered with clinical-grade dextrose (per oral, 1.5 g/kg), and blood glucose was monitored at 0, 10, 20, 30, 45, 60, 90, and 120 min after the sugar load. Almost all animals in the HFD group demonstrated more than 600 mg/dl blood glucose, and therefore the OGTT area under the curve could not be calculated for this group.

### Transthoracic Echocardiography and Hemodynamic Assessment

All the animals were subjected to transthoracic echocardiography on the penultimate day of the study using Vivo 770 high-resolution image systems (Visual Sonics, Toronto, Canada). Briefly, the animals were anesthetized with 2% isoflurane–oxygen mixture, and all ventricular and pulmonary dimensions and functions were recorded. Subsequently, invasive hemodynamic assessment was performed as explained previously (22). Following the hemodynamic measurement, the animals were euthanized, blood was withdrawn, a thoracotomy was performed to exsanguinate the heart and the lung, and finally, all other required organs were collected and tissue samples were processed for RNA, protein, and histology assessments. Right ventricular hypertrophy or Fulton's index [RVH = RV / (LV + S)] was calculated as the ratio of wet weight of the right ventricle (RV) to the left ventricle + intra-ventricular septum (LV + S). A complete blood count analysis was performed at Vanderbilt University Medical Center Pathology Core.

### Western Blot and Dot Blot Analyses

After euthanization, the superior lobe of the right lung was frozen for protein work and homogenized in radioimmunoprecipitation assay buffer supplemented with protease inhibitor cocktail (Sigma Aldrich, St. Louis, MO, USA). Following the protein estimation, an equal amount (30 μg) of protein was resolved on 4–12% NuPAGE gel (Thermo Fisher Scientific, Waltham, MA, USA) and transferred electrophoretically onto a polyvinylidene fluoride membrane, and the membranes were blocked with 5% non-fat milk solution in Tris-buffered saline with 0.1% Tween 20 for 1 h. Subsequently, the membranes were probed with the following antibodies: Caveolin (1:10,000), Caveolin 2 (1:500), Cavin 1 (1:1,000), EHD2 (1:1,000), PACSIN2 (1:1,000), and β-actin (1:5,000) overnight at 4°C. The blots were developed on the following day and visualized on the ChemiDoc MP imaging system (Bio-Rad Laboratories, Hercules, CA, USA). For the dot blot experiment, 200 μg of protein was used as per the manufacturer's instruction.

### Immunohistochemical Analysis

After euthanization, the left lobe of the lung was processed and paraffin-embedded, and immunohistochemical staining was performed as explained previously (23). CD45 (1:100), CD68 (1:100), and α-smooth muscle actin (1:200) antibodies were used for the assessment of macrophage and pulmonary vessel muscularization (24), and the images were analyzed as explained previously.

### Mass Spectrometry Identification of Mutant Cav1 in Mouse Tissues

Cav1 was immunoprecipitated from mouse tissues using Dynabeads® Protein G Immunoprecipitation Kit (Thermo Fisher Scientific, Waltham, MA, USA). Then, 5 μg pAb h1-97 IgG (20 μl) was cross-linked to 50 μl Dynabeads® Protein G by BS 3 (Sulfo-DSS; Thermo Scientific™ Pierce™) according to the manufacturer's suggested protocol. Tissues were lysed at 4°C with 1,000 μl CelLytic™ M buffer (Sigma-Aldrich), and the lysed cells were centrifuged for 15 min at 12,000 × *g* at 4°C to pellet the cellular debris. The protein-containing supernatant was transferred into a new tube containing Dynabeads®-Ab complex and incubated with rotation for 10 min at room temperature. The tube was placed on a magnet, and the supernatant (“unbound”) was transferred to a clean tube for further analysis. The Dynabeads®-Ab-antigen complex was washed four times, using 200 μl washing buffer for each wash. The final wash was conducted in a new clean tube. The wash solution (“wash”) was collected for further analysis. The Dynabeads®-Ab-antigen complex was resuspended with 20 μl elution buffer, 10 μl premixed NuPAGE® LDS sample buffer, and NuPAGE sample reducing agent and heated for 10 min at 70°C. The supernatant was loaded onto the gel for western blot and mass spectrometry identification. Bands corresponding to caveolin-1 were excised and subjected to in-gel trypsin digestion. The resulting peptides were analyzed by a 70-min data-dependent liquid chromatography–tandem mass spectrometry (MS/MS) analysis. Briefly, the peptides were autosampled onto a 200 × 0.1-mm (Jupiter 3 μm, 300 A) self-packed analytical column coupled directly to an LTQ (ThermoFisher) using a nanoelectrospray source and resolved using an aqueous-to-organic gradient. A series of full scans followed by five data-dependent MS/MS was collected throughout the separation. Dynamic 17 exclusion was enabled to minimize the acquisition of redundant peptide spectra. MS/MS spectra were searched *via* SEQUEST against a mouse database (UniprotKB—reference proteome set) to which the mutant caveolin sequence had been appended and that also contained a reversed version for each of the entries (25). The identifications were filtered and collated at the protein level using Scaffold (Proteome Software).

### Statistics

Graph Pad Prism, version 8.4.2 (La Jolla, CA, USA) was used for the statistical analysis. One-way ANOVA data analysis followed by Bonferroni's test for multiple comparison was performed for end-point parameters. Two-way ANOVA followed by Tukey's multiple-comparison *post-hoc* analysis was performed to compare the interaction between genotype and treatment. Pearson correlation coefficient was performed to compare the correlation between variables. The values are represented as means ± SEM; *p* ≤ 0.05 were considered as statistically significant.

## Results

### Creation of the Cav1^P158Pfsx22^ Mice

Although Caveolae have been studied in the context of PAH for many years, it was studied in the context of knock-out (7–10). Before 2012, CAV1 mutations had never been identified as causal in human PAH. The initial mutations identified, a P158P fsX22 mutation, was not obviously a complete loss-of-function mutation. The mutation impacted only the final 20 amino acids, replacing the existing termination of the protein with a new rather different termination of 21 amino acids.

**Figure 1:**
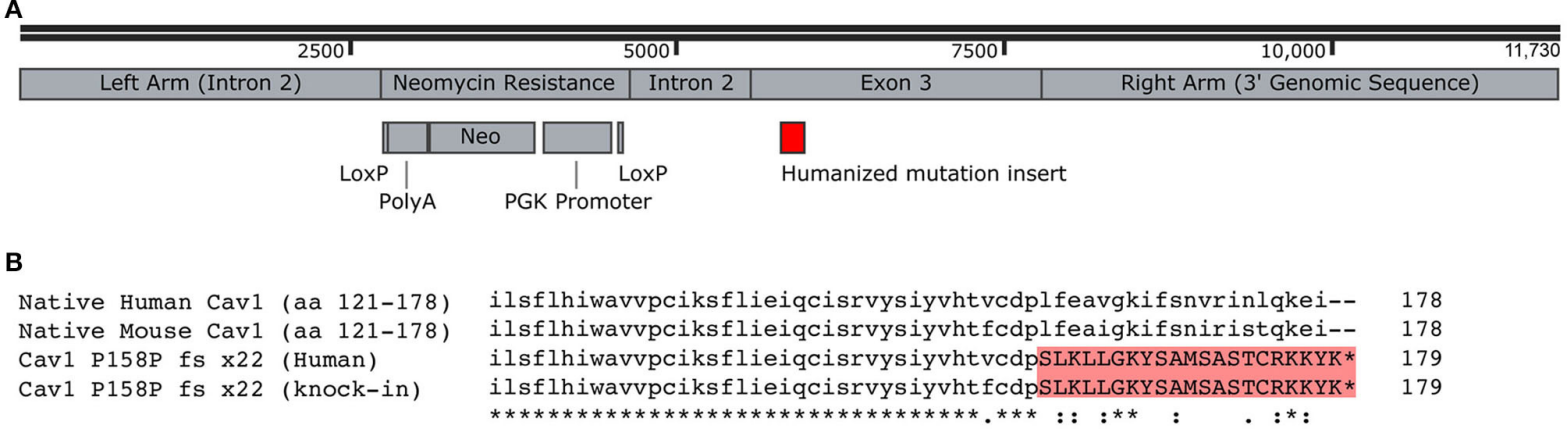
Creation of Cav1^P15PfsX22^ mice. **(A)** Recombination construct used for homologous recombination in embryonic stem cells. The LoxP sites were recombined, removing the neomycin resistance, before the creation of mice. **(B)** Alignment of human and mouse C-termini of Cav1, demonstrating the addition of a novel termination sequence with the human mutation and the inclusion of an identical sequence in the murine knock-in. The asterisk denotes identity, the dot denotes a semi-conservative substitution, and the colon denotes a conservative substitution.

Our goal here was to study the physiologic impact of the human CAV1 mutations as closely as possible in a mouse model. Although the mouse and the human CAV1 sequences are very well-conserved—both are 178 amino acids, with 169/178 amino acids identical (95%) and 175/178 conserved—third base redundancy means that just inserting the frameshift would have produced a different amino acid sequence in mice. We thus replaced the end of the mouse CAV1 with the mutated human sequence rather than merely inserting the frameshift (Figure 1A). The final amino acid sequence of our knock-in of the human mutation into the mouse is depicted in Figure 1B.

These Cav1^P15PfsX22^ mice were created through routine embryonic stem cell methods with homologous recombination. The resulting mice were born with normal Mendelian proportions and were apparently healthy and bred well, even as homozygous.

### Cav1^P15PfsX22^ Protein Is Largely Degraded in Mice, With an Organ-Specific Pattern of Loss of Accessory Proteins

First, we examined the expression of both Cav1 protein in knock-in mice as well as with other proteins which are part of caveolae. These include Cav2, a homolog with which Cav1 normally hetero-oligomerizes, Cavin1, a protein that stabilizes caveolae, and EHD2 and PACSIN, which form an actin cytoskeleton-interacting complex as part of caveolae (26).

**Figure 2:**
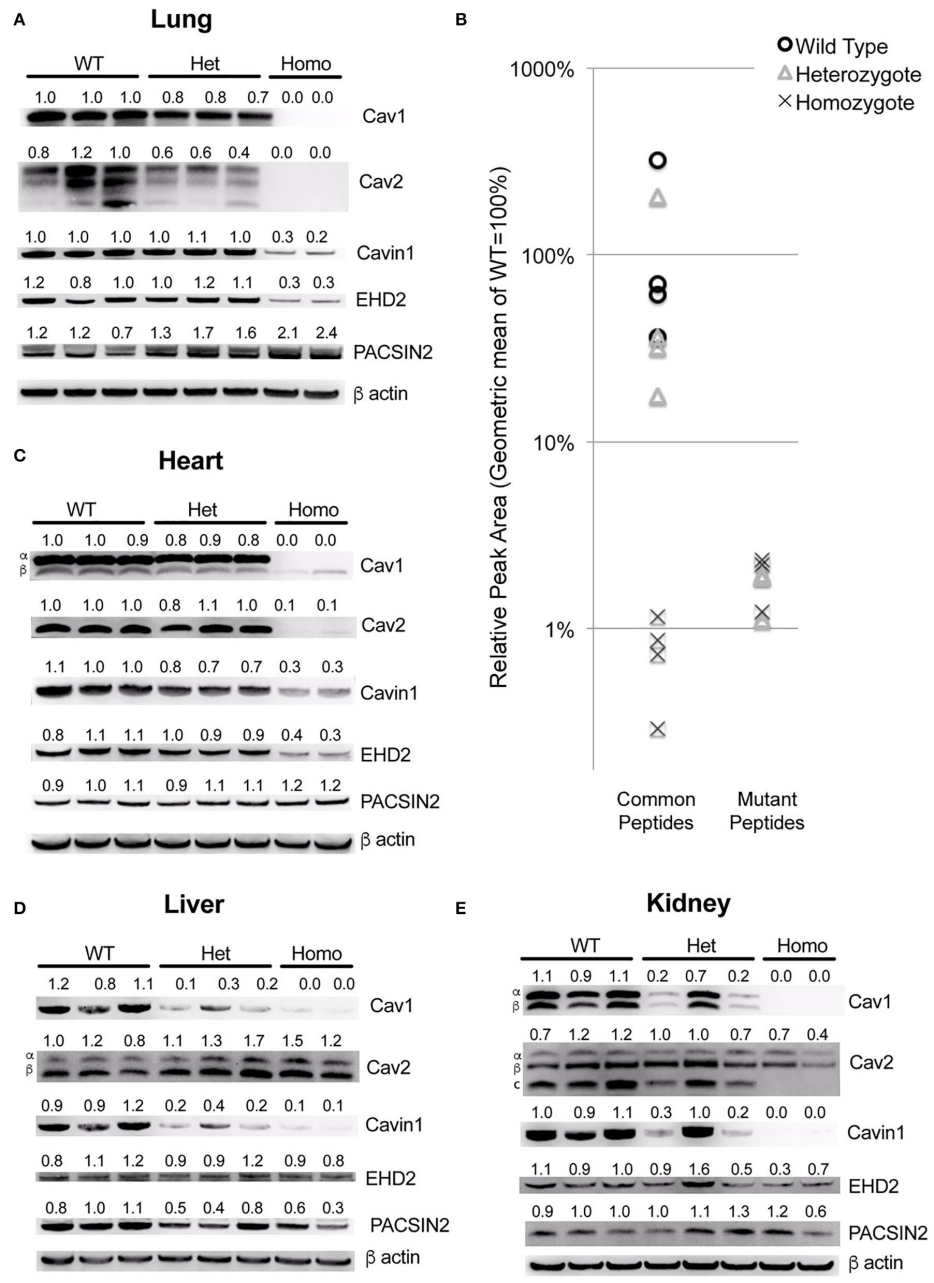
The Cav1^P15PfsX22^ protein is largely degraded in mice, with an organ-specific pattern of loss of accessory proteins. **(A)** Western blots for the indicated proteins from whole mouse lung from wild-type, heterozygous and homozygous mice for the Cav1^P15PfsX22^ mutation—the numbers indicate densitometry normalized to wild type and to beta-actin. **(B)** The mass spectrometry quantification results of proteins from wild-type, heterozygous, and homozygous mice suggest that very little mutant protein is retained even in heterozygous mice. **(C)** Western blots for the indicated proteins from whole mouse heart. **(D)** Western blots for the indicated proteins from whole mouse liver. **(E)** Western blots for the indicated proteins from whole mouse kidney.

By quantitative RT-PCR using primers that spanned exons 2 and 3, we found that the spliced RNA expression of the knock-in was comparable to that of the wild-type, as expected (not shown). However, in the lungs, we found that the overall Cav1 levels were reduced by about 20% in heterozygous animals and undetectable in homozygous ones (Figure 2A). The levels of Cav2 were decreased at approximately the same extent as Cav1, in accord with prior literature indicating that the expression of Cav1 is required for the stability of Cav2 (27, 28). We also saw a decrease in accessory proteins EHD2 and Cavin1, but not PACSIN2, which increased as Cav1 decreased.

Since the native and the mutant proteins are of the same size, and the antibody recognizes both (there is no antibody to the mutant section of the protein, and we did not tag it in an attempt to preserve the function), we could not readily determine how much of this was from the native and how much was from the mutant protein. Although the homozygous animal had no visible Cav1 band by western blot, this is presumably because it is retained in the ER and degraded, as has been previously reported (17, 18). However, at least when overexpressed, it has been reported that native Cav1, by complexing with a mutant, can assist with the transport to caveolae, which implies that perhaps the mutant might be stabilized in the heterozygous mice. We thus assessed the levels of mutant compared to wild-type protein by using mass spectrometry. We assessed the levels of four different peptides common between the mutant and the wild-type versions of Cav1 and three different peptides specific to the mutant version. We found that, even in heterozygous animals, the expression of the mutant peptides was very low—on average only 1.4% of that of the wild-type Cav1 (Figure 2B).

The pattern in other organs was distinctive—in the heart, most proteins were similar to the lung, but there was no increase in PACSIN2 levels in the homozygous animals (Figure 2C). In the liver, there was no decrease in Cav2 or EHD2, but Cavin1 was still decreased and PACSIN2 appeared to trend down (Figure 2D). The kidney was similar to the liver, except that Cav2 decreased moderately in the homozygous animals (Figure 2E). This preservation of Cav2 in some tissues when Cav1 is missing contradicts some literature but is supported by others, which have found that Cav2 can form homo-oligomers (29). Broadly, these data suggest that, in all circumstances, the mutant Cav1 is rapidly degraded, and the effect on accessory proteins is very organ specific.

### Initial Phenotyping of Cav1^P158Pfsx22^ Mice Showed a Moderate Phenotype Consistent With Prior Reports on Cav1 Knockout Mice

**Figure 3:**
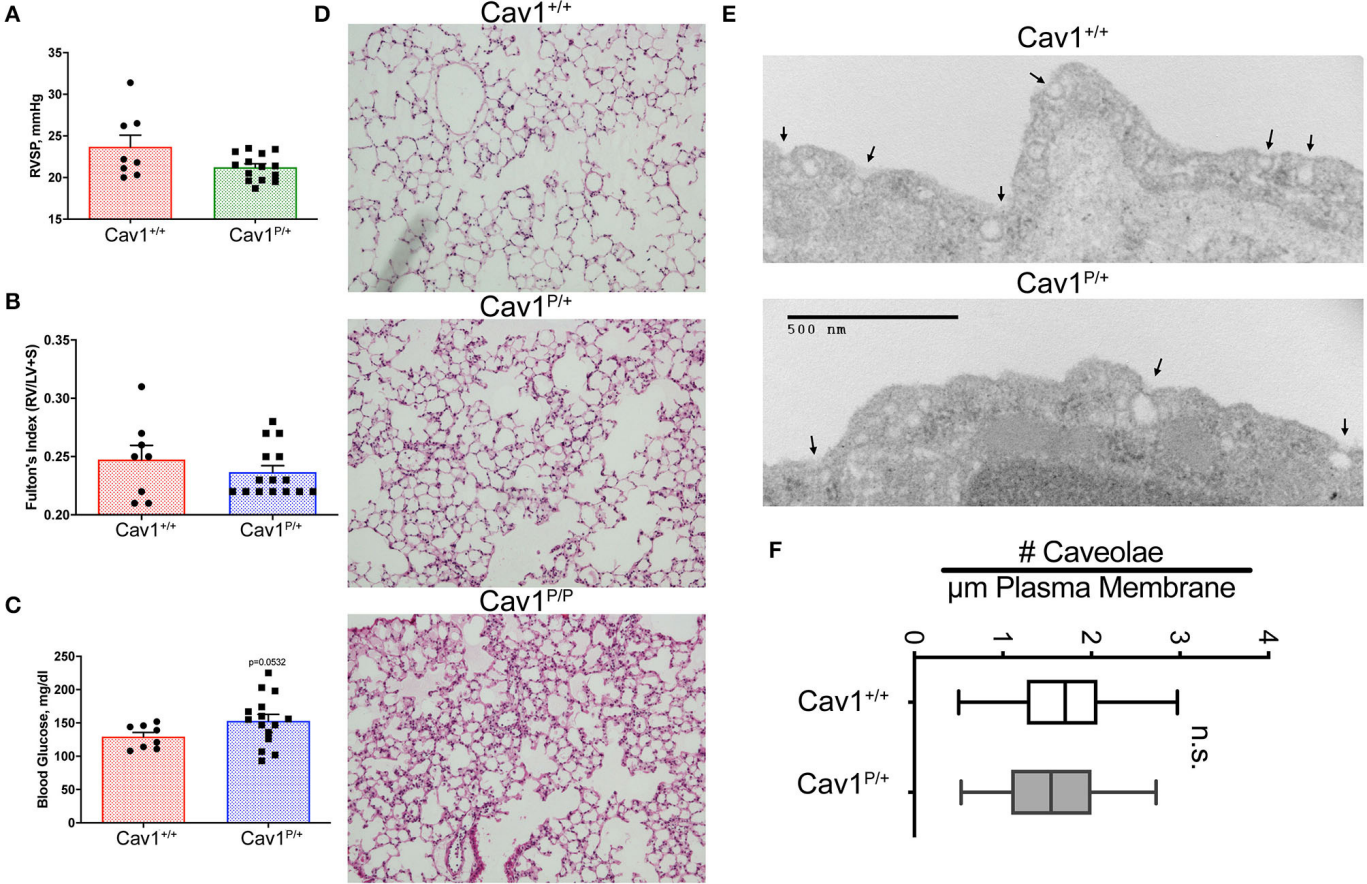
Cav1^P15PfsX22^ mice have a mild phenotype, with no change in the number of caveolae in heterozygous mice. No difference in **(A)** right ventricular systolic pressure or **(B)** right ventricle hypertrophy, and only subtle differences in **(C)** blood glucose in older heterozygous (27–30 weeks) Cav1 mutant mice. **(D)** Some Cav1 mutant animals appeared to have an increased number of pulmonary inflammatory cells, but the phenotype was inconsistent. Lung architecture was observed under × 100 magnification. **(E)** Example of transmission electron microscopy of wild-type (top) and heterozygous (bottom) Cav1 mutants. **(F)** Based on counts of 30–38 fields from three separate mice of each genotype, there is no significant difference in the number of caveolae between wild-type and heterozygous endothelium for Cav1^P15PfsX22^ (referred to as Cav1^P^ in the figure).

As an initial examination of the phenotype in these mice, we performed hemodynamic and histologic phenotyping on 27- to 30-weeks-old heterozygous Cav1 mutant and littermate control animals. The heterozygous were our focus because the heterozygous mutation results in pulmonary arterial hypertension in human patients. We found no significant difference in right ventricular systolic pressure (RVSP) (Figure 3A) or right ventricular hypertrophy (Figure 3B) and only a slight trend toward increased blood glucose (Figure 3C, *p* = 0.0532, mean 153 vs. 129 mg/dl). The results in a smaller experiment with younger animals (8- to 10-weeks-old mice) which included a few homozygous animals showed a slight increase in RVSP in homozygous of about 30 mmHg, consistent with previous reports (Supplemental Figure 1A), but neither Fulton index (right ventricular hypertrophy) nor blood glucose changed in the mutant mice (Supplemental Figures 1B,C). By histology, the Cav1 mutant animals (Cav1^P158Pfsx22^, hereafter referred to as Cav1^P^), appeared to have increased inflammatory cell burden in the lungs (Figure 3D, Supplemental Figure 2 for higher-resolution pictures), but this pattern was inconsistent and not apparent in the older mice initially. We performed a transmission electron micrography of lung sections and found no difference in the number of caveolae between wild-type and heterozygous endothelium (Figures 3E,F).

### Stressing Cav1^P158Pfsx22^ Mice With a Diabetes Model Results in Only Subtle Changes in Hemodynamic Phenotype

Cav1 mutation in humans is more commonly associated with lipodystrophy, and Cav1 knockout in mice has previously been shown to cause broader metabolic dysfunctions (30), in addition to a failure to gain weight under a high-fat diet (31). We thus hypothesized that the pulmonary phenotype might be more strongly brought out by metabolically stressing the mice. We used a high-fat diet with streptozotocin (STZ) to metabolically stress the Cav1^P158Pfsx22^ mice. STZ is a naturally occurring alkylating agent which is extremely toxic to insulin-producing beta cells; two doses of STZ should thus result in insulin deficiency, whereas the high-fat diet results in insulin resistance.

**Figure 4:**
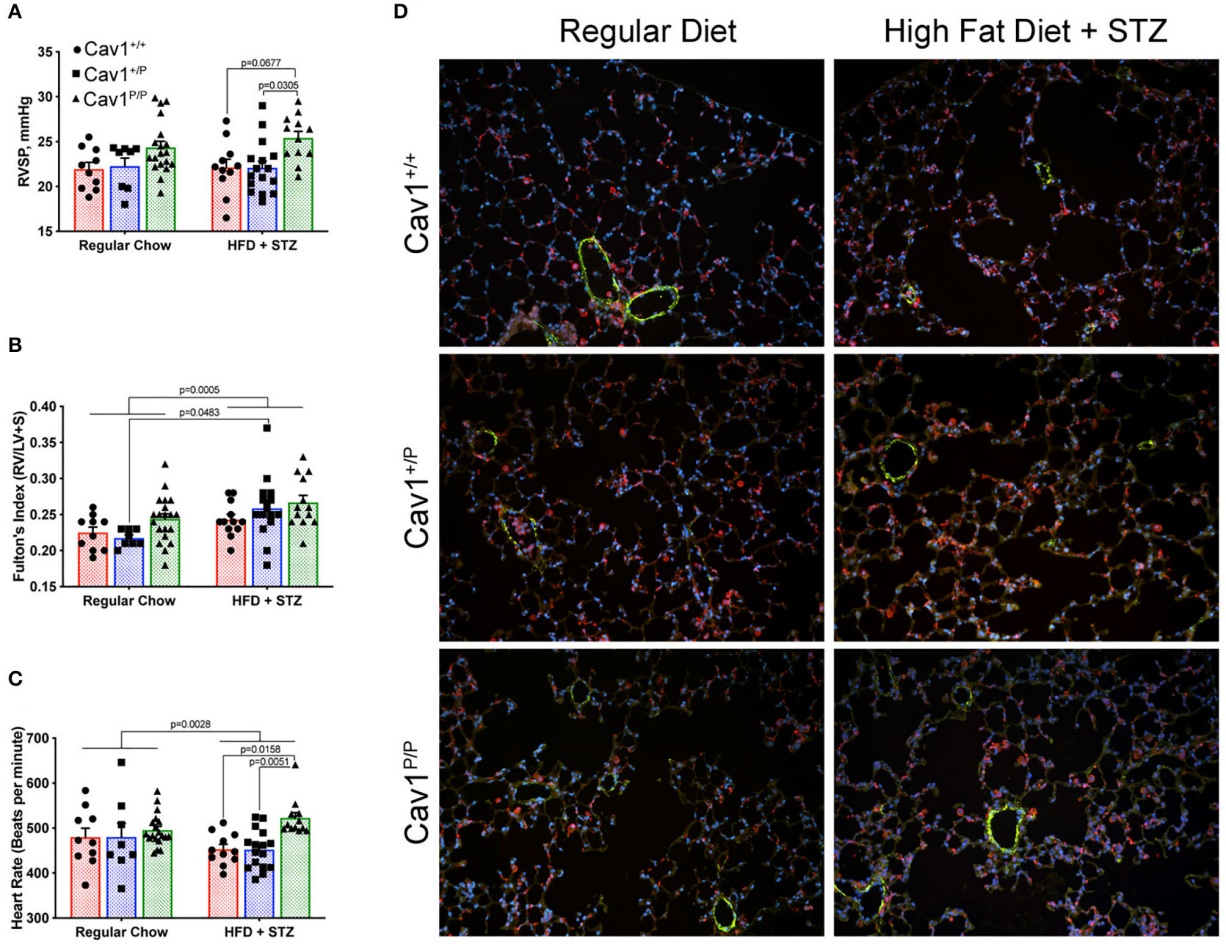
A diabetes model, high-fat diet plus streptozotocin (HFD + STZ), does not bring out a more dramatic hemodynamic phenotype in Cav1^P15PfsX22^ mice. **(A)** Right ventricular systolic pressure was not changed by diet; Cav1 homozygous mutant mice (Cav1^P/P^) showed a slight increase in pressure overall. **(B)** Right ventricular hypertrophy was slightly higher in HFD + STZ mice, but this was not genotype dependent. **(C)** Heart rate under anesthesia was slightly altered by HFD + STZ as shown. **(D)** Lung histology was fairly normal in all groups. DAPI (blue), smooth muscle actin (green), and CD45 (red) immunofluorescence. Pulmonary vasculature was observed under × 200 magnification.

The inclusion of high-fat diet plus streptozotocin (HFD + STZ) made a little difference to the hemodynamics. Although the mice homozygous for mutation once again had slightly higher pressures, the increase was lower than what we had previously seen in younger mice; HFD + STZ did not cause meaningful changes in the pressures of any group (Figure 4A). HFD + STZ slightly increased RV mass across groups—there was no genotype-specific effect (Figure 4B). There were small but statistically different changes in heart rate under anesthesia, with the control and the heterozygous mice moving downwards slightly and the homozygous mice moving upwards slightly (Figure 4C), but the changes probably are not large enough to be meaningful. The lung histology across groups was fairly normal (Figure 4D)—these pressures are not high enough to drive muscularization (see Supplemental Figure 3 for detailed counts of muscularized vessels, which did not change). We also observed a slight increase in CD45^+^ inflammatory cells in the homozygous group, but it was too patchy within and between mice to be statistically significant (not shown).

We also measured a large number of cardiac indices—left ventricular fractional shortening, left ventricular ejection fraction, left ventricular cardiac output, cardiac index, pulmonary artery area, right ventricular cardiac output, and right ventricle internal dimension during diastole. There were no meaningful differences between groups in any of these measurements (not shown).

### Cav1^P158Pfsx22^ Homozygous Mice Have Altered Body Composition and Glucose Tolerance Compared to Wild-Type or Heterozygous Mice

**Figure 5:**
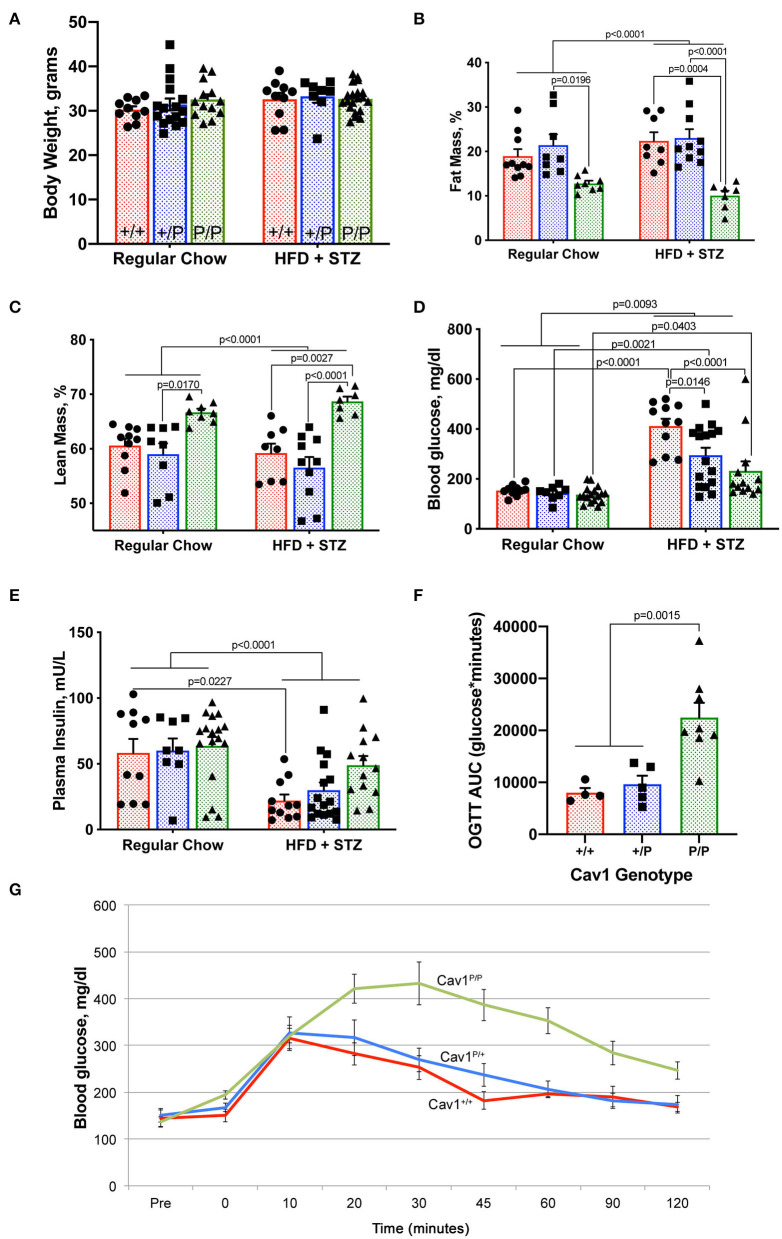
Cav1^P158Pfsx22^ homozygous mice exhibit altered body composition and glucose tolerance. For all figure parts, red shading/circles indicate wild-type mice, blue shading/squares indicate heterozygous mice, and green shading/triangle indicates homozygous mice. Each symbol is the measurement from one animal, with the bar showing mean and SEM, except for **(G)**. Statistics were performed by ANOVA with *post-hoc* tests. **(A)** Body weight is not impacted by genotype or diet. **(B)** Homozygous mice have a lower percentage of body fat. **(C)** Homozygous mice have a higher percentage lean mass. **(D)** Although blood glucose is increased significantly in all groups with high-fat diet plus streptozotocin (HFD + STZ), it is increased to a lower extent in homozygous Cav1^P15PfsX22^ mice. **(E)** Insulin is reduced in HFD + STZ mice but to a lesser extent in Cav1 homozygous mice. **(F)** Although they can produce more insulin, homozygous mice take a much longer time to clear oral glucose. OGTT, oral glucose tolerance test; AUC, area under the curve. These are regular chow-fed mice (HFD + STZ are off-scale for OGTT). **(G)** Average blood glucose at each time point during the oral glucose tolerance test by genotype. The error bars are standard error of the mean.

Mice on either regular diet or high-fat diet plus streptozotocin were subjected to metabolic phenotyping. Although mice on high-fat diet alone tend to have an increased body weight, HFD + STZ did not result in significant differences in body mass, either by diet or by genotype (Figure 5A). However, mice on HFD + STZ had a slightly higher body fat on average, except for Cav1^P/P^ mice, which had a significantly lower proportion of body fat and a higher proportion of lean mass (Figures 5B,C) at the same overall weight. Caveolae are abundant in adipose tissues, and adipose tissues in Cav1 knockout mice lack perilipin, which is necessary to stabilize adipocyte oil droplets (32). In contrast, caveolae in the skeletal muscle is produced by Cav3 (28), whose action is independent of Cav1 and thus likely still functional in these mice. These findings are thus consistent with the literature.

By measurements of blood glucose (Figure 5D) and plasma insulin (Figure 5E), we found that the Cav1^P/P^ mice retained greater insulin production than the other genotypes. It is possible that streptozotocin is reliant on caveolae for its function. The heterozygous mice appeared to have an intermediate phenotype. Cav1^P/P^ mice had a significantly delayed glucose clearance, with the area under the curve of the oral glucose tolerance test being nearly three times as high in homozygous as in heterozygous or wild-type mice (Figures 5F,G). The data shown are from regular chow-fed animals; OGTT was not possible in HFD + STZ mice because their glucose readings were higher than the measurable level throughout the first 90 min of the OGTT test, regardless of genotype. This lower insulin sensitivity is consistent with earlier studies on caveolin 1, which directly regulates insulin receptors (33, 34).

### Cav1^P158Pfsx22^ Mice Have Much Lower Waking Locomotion but Are Similar to Wild-Type in Energy Balance Measurements

**Figure 6:**
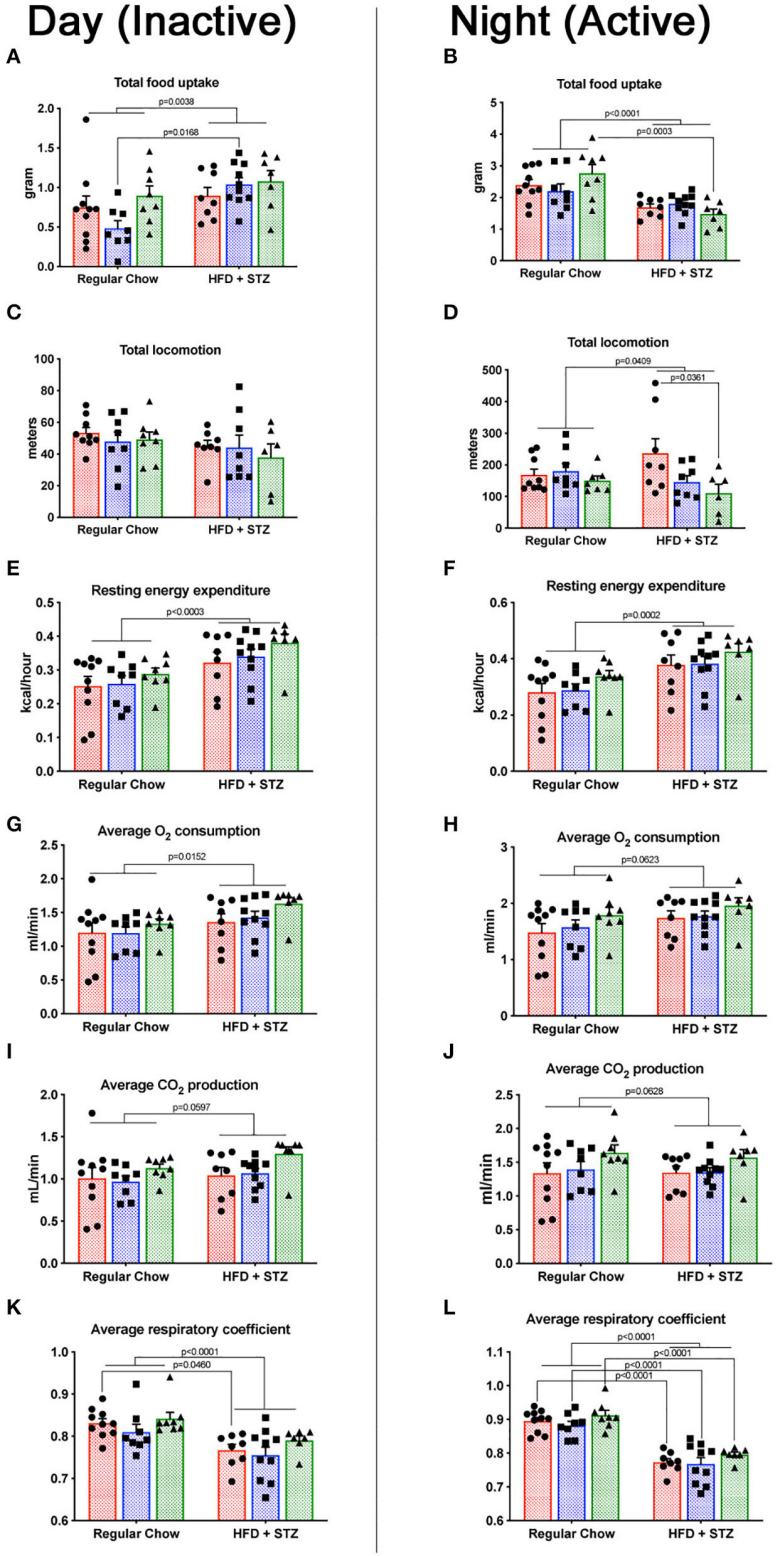
Cav1^P158Pfsx22^ mice have lower waking locomotion but are similar to wild-type mice in energy balance measurements. For all figure parts, red shading/circles indicate wild-type mice, blue shading/squares indicate heterozygous mice, and green shading/triangles indicate homozygous mice. Each symbol is the measurement from one animal, with the bar showing the mean and SEM. The left half of the figure **(A,C,E,G,I,K)** are measurements made during the day, when mice were inactive; the right half of the figure **(B,D,F,H,J,L)** are measurements made during the night, when mice were active. **(A,B)** Food consumed during the day and the night, respectively. **(C,D)** Total locomotion (this includes climbing as well as horizontal locomotion but is nearly identical to horizontal locomotion alone. **(E,F)** Resting energy expenditure is significantly higher with high-fat diet plus streptozotocin (HFD + STZ) in both day and night. **(G,H)** The oxygen consumption trends were higher. **(I,J)** CO_2_ production. **(K,L)** The respiratory coefficient is significantly lower in HFD + STZ, as expected.

We measured a variety of metabolic parameters in heterozygous and homozygous Cav1^P158Pfsx22^ mice, both during the night (waking) and during the day (sleeping). These include food uptake, locomotion, energy expenditure, O_2_ consumption, CO_2_ production, and respiratory coefficient (Figure 6). For most of these, there was a diet-specific effect but no genotype-specific effect. The exception to this was locomotion—homozygous Cav1^P158Pfsx22^ mice move less than half as much on average as wild-type mice during their waking hours (Figure 6D). The heterozygous mice were intermediate—this is one of only a small number of parameters in which the heterozygous mice show any difference compared to the wild-type mice. This is consistent with previously published results, in which Cav1 knockout mice had reduced endurance in a forced swim test (27); however, the current data indicate that this reduced physical ability is true of normal activity as well.

The respiratory coefficient, the ratio of CO_2_ exhaled to O_2_ consumed, is indicative of the preferred metabolic substrate, with pure carbohydrate metabolism producing a number of “1” and pure fat metabolism producing a number of “0.7.” That similarity of respiratory coefficient between homozygous and wild-type animals (Figures 6K,L) is moderately unexpected—cav1 depletion might be expected to interfere only with certain metabolic pathways. In shifting to a high-fat diet, though, it appears that Cav1^P158Pfsx22^ mice shift to a higher proportion of fat burning in the same proportion as wild-type mice.

### Mice Homozygous for the Cav1^P158Pfsx22^ Mutation Have Elevated Circulating Monocytes and Elevated Monocyte Chemoattractant Proteins in the Lungs

**Figure 7:**
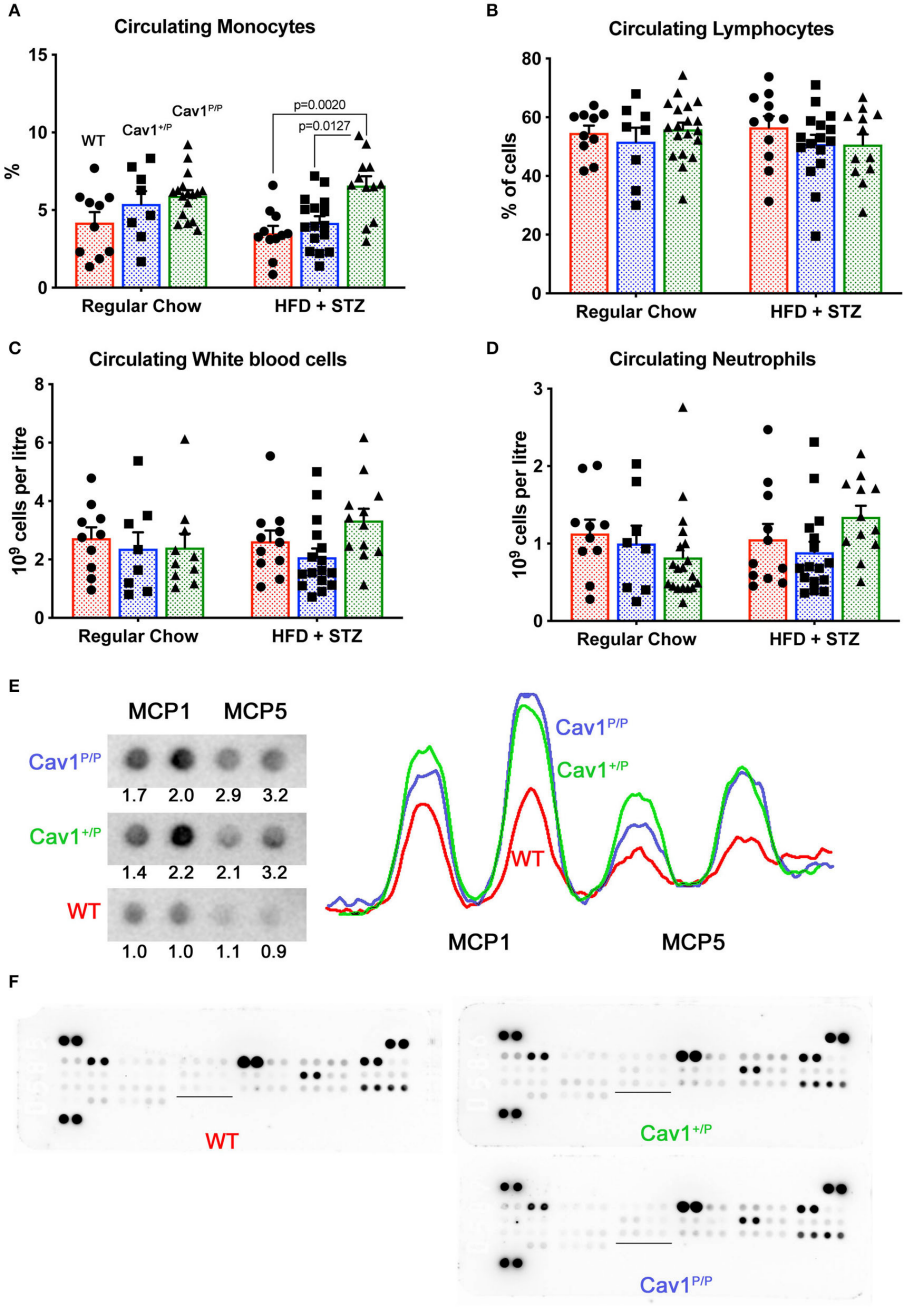
Mice homozygous for the Cav1^P158Pfsx22^ mutation have elevated circulating monocytes and elevated monocyte chemoattractant proteins in the lungs. For figure parts **(A–D)**, red shading/circles indicate wild-type (WT) mice, blue shading/squares indicate heterozygous mice, and green shading/triangles indicate homozygous mice. Each symbol is the measurement from one animal, with the bar showing the mean and SEM. The statistics are derived by ANOVA, with *post-hoc* tests to compare individual groups. **(A)** The percentage of circulating monocytes is increased in homozygous Cav1 mutant animals, although diet has no significant effect. **(B)** The percentage of circulating lymphocytes is not altered by genotype or diet. **(C)** White blood count is not altered by genotype or diet. **(D)** Circulating neutrophils are not altered by genotype or diet. **(E)** MCP1 and MCP5 protein are increased in both heterozygous and homozygous mutant animals. The tracing is densitometry for each of the blots at the left; the numbers are areas under each curve, normalized to the average WT level of each protein. **(F)** Overall cytokine blots on lung proteins from WT, heterozygous, and homozygous mutants have few differences aside from MCP1 and MCP5 (the underlined section is blown up in **(E)** above).

We performed complete blood counts on mice from the dietary study (Figure 7). We found that circulating monocytes were increased in homozygous Cav1^P158Pfsx22^ mice overall, but without a clear effect of diet (Figure 7A). In contrast, there were no significant changes in lymphocytes, overall white blood count, or neutrophils (Figures 7B–D). We tested the cytokine levels in plasma and the lung by dot blot array. Although we found no significant differences in cytokines in plasma (not shown), we found increases in monocyte chemoattractant proteins 1 and 5 (MCP1/CCL2, MCP5/CCL12) in the lung (Figure 7E). There were otherwise few differences between groups (Figure 7F). There are a few previous publications linking Cav1 to monocyte attraction or function (35, 36), but this is not typically thought of as a primary role for caveolae.

These data, combined with the trend toward increased CD45^+^ cells that we saw in previous figures (Figure 4D), suggest that Cav1^P158Pfsx22^ mutation in homozygous mice is associated with moderate infiltration of inflammatory cells in the lung, but more specifically the circulating monocytes. CD68 staining for monocytes/macrophages found a trend toward an increase in the lungs of Cav1 mutant mice, but the level of variability was too high at the current *n* for it to be significant (Supplemental Figures 4A,B). In particular, there were frequently large numbers immediately surrounding large vessels (Supplemental Figure 4C).

### Heterozygous Cav1^P158Pfsx22^ Mice Do Not Appear to Have a Hemodynamic Susceptibility to LPS but May Have a Susceptibility to Body Weight

The trends in histology shown in Figures 3, 4, combined with the mild suggestion of increased circulating monocytes and increased lung monocyte chemoattractant proteins in Figure 7, made us wonder if the Cav1^P158Pfsx22^ mice would be particularly susceptible to an inflammatory insult. We thus performed a small study using low-dose intratracheal installation of LPS, focusing on heterozygous animals, since human patients with this mutation are heterozygous.

**Figure 8:**
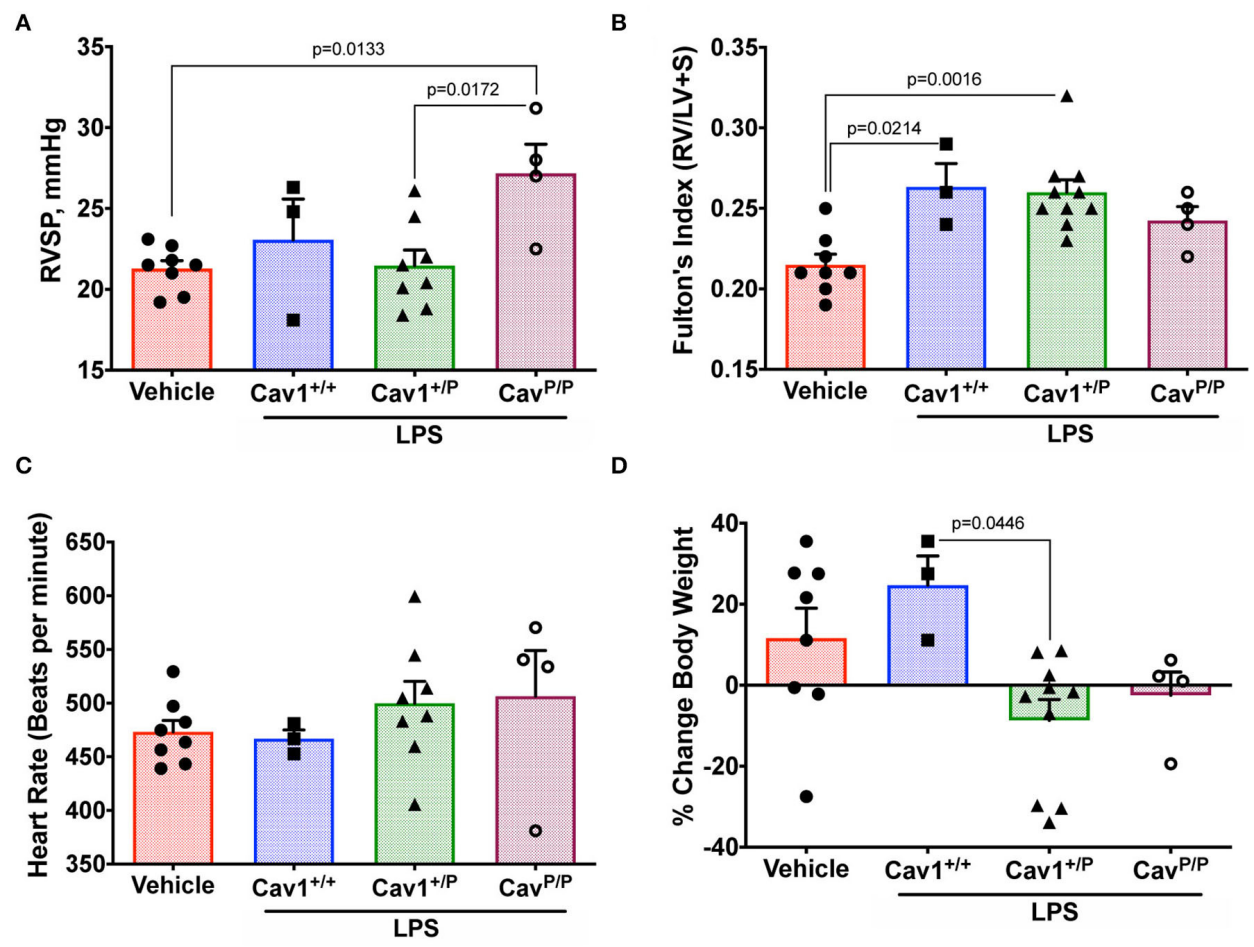
Heterozygous Cav1^P158Pfsx22^ mice do not appear to have a hemodynamic susceptibility to lipopolysaccharide (LPS) but may have a susceptibility to body weight. For all figure parts, blue shading/squares indicate wild-type mice, green shading/triangles indicate heterozygous mice, and maroon shading/open circle indicate homozygous mice in the LPS group. Red shading/circles in vehicle control mice indicate a mixture of genotypes (three wild type, three heterozygous, and two homozygous mice). Each symbol is the measurement from one animal, with the bar showing the mean and SEM. The mice given a chronic low-dose of LPS through installation for 3 weeks had **(A)** no changes in right ventricular systolic pressure (compared to Figure 4A). **(B)** No genotype-dependent changes in right heart hypertrophy. **(C)** No differences in heart rate under anesthesia. **(D)** Heterozygous animals may be sensitive to LPS in weight gain, by ANOVA.

We utilized a low dose of LPS with repeated dosing, which we had previously determined in other models to have a little effect on wild-type mice but which brought out a phenotype in susceptible animals (37). It did not, however, appear to have an impact on RVSP in either heterozygous or homozygous mice (Figure 8A—compare to Figure 4A). Fulton index is a little higher in all LPS-treated animals, but this did not differ by genotype (Figure 8B). Heart rate under anesthesia was similar across groups (Figure 8C). The only element here in which there was an apparent susceptibility was body weight; we would have anticipated that body weight would increase after 3 weeks on breeder chow, and it generally did for WT mice. However, the weight stayed the same or decreased in LPS-treated heterozygous mice (Figure 8D). By ANOVA, genotype was significant at *p* = 0.0446.

## Discussion

This study established that the new human CAV1 termination sequence, when knocked in to the native locus in mice, results in the degradation of a large majority of mutant proteins, in both heterozygous and homozygous animals, resulting in the loss of CAV2 and accessory proteins in some, but not all, tissues (Figure 2). Heterozygous mutants have no change in their hemodynamic phenotype, and their number of caveolae in the pulmonary vascular endothelium is unchanged (Figure 3)—there appears to be a compensatory upregulation of wild-type Cav1. Furthermore, they have no particular hemodynamic susceptibility to either metabolic challenge (Figure 4) or inflammatory challenge (Figure 8). For most energy balance measurements, the heterozygous mice were more similar to the wild-type mice rather than to the homozygous—they did not share the homozygous increase in lean mass or their poor oral glucose tolerance (Figure 5). In other aspects, though, the heterozygous animals did show interesting differences. They appeared to have an intermediate phenotype in blood glucose and plasma insulin between wild-type and homozygous mice (Figures 5D,E). This was also the first study to show that the loss of Cav1 results in reduced spontaneous locomotion; the heterozygous mice appeared to have an intermediate phenotype (Figure 6D). Finally, the heterozygous Cav1 mutants appeared to have an intermediate phenotype in the increase in circulating monocytes seen in homozygous animals (Figure 7A), and they had the same increase in monocyte chemoattractant proteins (Figure 7E). While the LPS challenge did not increase their right ventricular systolic pressure, it did appear to make them susceptible to weight loss (Figure 8D).

The substantial degradation of the mutant protein in the homozygous mice is consistent with prior findings in cell culture that this mutation causes the protein to accumulate in the endoplasmic reticulum as the result of the introduction of an ER retention signal (17). Notably, the levels of Cav1 protein detected in heterozygous animals varied across tissues; they were only modestly decreased in the lung and the liver, whereas substantially less Cav1 was detected in the liver and the kidney. This suggests that in some tissues the wild-type copy of Cav1 may assist in the translocation of the mutant copy to the plasma membrane, as has been observed in cell culture studies (17, 18). Alternatively, the compensatory upregulation of WT Cav1 may occur in a tissue- or cell type-specific manner.

The metabolic findings in this study are largely supportive of the existing literature on the effects of the loss of Cav1. Both the lower fat mass (32) and the increased lean mass (28) are consistent with prior reports in mice and with the known association with lipodystrophy in human patients (4). The poor insulin sensitivity and the oral glucose tolerance AUCs are also consistent with earlier reports (33, 34). The previous publication showing that Cav1 knockout mice had difficulty with the forced swim test (27) makes sense because of their reduced ability to rapidly internalize glucose; the present study is the first to show that this is true even for a routine activity—in homozygous and, to a lesser extent, in heterozygous animals. The mechanism is presumably the same.

The inflammatory phenotype that we observed, while mild, is particularly interesting in the context of a recent publication in which reciprocal bone marrow transplants between wild-type and Cav1^−/−^ animals suggested that the pulmonary hypertensive phenotype of Cav1^−/−^ was being driven by circulating cells (38). We are not the first to see an association between macrophages and Cav1. Cav1 knockouts showed increased adventitial macrophages in aortic transplants (39). The overexpression of Cav1 reduced the infiltration of monocytes/macrophages in a mouse model of pulmonary fibrosis (40). Reduced Cav1 levels in monocytes specifically lead to increased chemotaxis activity (36). Furthermore, the loss of Cav1 in psoriatic skin as well as peripheral blood mononuclear cells and CD14^+^ monocytes results in skin inflammation and an increase in monocyte–macrophage lineage activities (36). In scleroderma patients, the decreased levels of Cav1 serve as a risk factor for the differentiation of monocytes to spindle-shaped fibrocytes (41). Potentially, these and other studies suggest that the loss of Cav1 may have a direct impact on monocyte/macrophage recruitment, function, or both.

Even the homozygous mice in the present study had less of a pulmonary hypertensive phenotype than had previously been published for Cav1 knockouts, although the level of pulmonary hypertension in other studies has also been variable, with some studies showing no increased pressure at baseline (42). These differences are probably just strain differences—the original knockouts were created on a mixed 1295SVJ × Black Swiss background, whereas ours were on a mixed C57/Bl6J × FVB/N background. Differences in background genotype can make a large difference in the pulmonary hypertensive phenotype. The same is true, really, of the patients. Mutations that seem functionally similar in other individuals produce lipodystrophy, not pulmonary hypertension, which makes it seem likely that the reason that we did not see pulmonary hypertension in these mice is that they lacked the underlying polymorphisms associated with disease in the patients, although it could be due to differences between human and mouse anatomy or Cav1 function.

Hypoxia is a standard “second hit” for pulmonary hypertension genetic models, and Cav1 has previously been extensively associated with vasoreactivity, particularly through nitric oxide (10). However, the susceptibility of Cav1 knockout mice to hypoxia has been previously published, with results suggesting no particular pulmonary susceptibility but an increased rate of right ventricular failure (42). Because this model is likely to be molecularly similar to knockouts (Figure 2), we did not pursue susceptibility to hypoxia in this model.

Whether the inflammatory or the metabolic abnormalities seen in these mice are actually drivers of the hemodynamic phenotype is currently unknown. However, these defects are causally associated in some other models, and so overall, the present work adds to the body of literature associating insulin resistance and increased inflammation with pulmonary hypertension.

## Data Availability Statement

The raw data supporting the conclusions of this article will be made available by the authors, without undue reservation.

## Ethics Statement

The animal study was reviewed and approved by Institutional Animal Care and Use Committee at the Vanderbilt University Medical Center.

## Author Contributions

AK, AH, AR, CC, EA, EC, and JW conceived and designed the experiment. AC, AR, CC, CM, SG, and SS performed the experiment. AR, AK, AH, CC, and JW analyzed the data. AR and JW wrote the paper. All authors contributed to the article and approved the submitted version.

## Conflict of Interest

The authors declare that the research was conducted in the absence of any commercial or financial relationships that could be construed as a potential conflict of interest.

## Abbreviations

AUC: area under the curve
CAV1: caveolin-1
CAV2: caveolin-2
CAVIN1: caveolae-associated protein 1
ECHO: echocardiography
EDA: end diastolic area
EF: ejection fraction
EHD2: EH domain containing 2
ER: endoplasmic reticulum
HFD: high-fat diet
IPF: idiopathic pulmonary fibrosis
LPS: lipopolysaccharides (from bacteria)
MCP-1: monocyte chemoattractant protein 1
MCP-5: monocyte chemoattractant protein 5
PACSIN2: protein kinase C and casein kinase substrate in neurons 2
PAH: pulmonary arterial hypertension
PH: pulmonary hypertension
RVH: right ventricular hypertrophy
RVSP: right ventricular systolic pressure
STZ: streptozotocin.
