# Supplementary material for: Expression of a Human Caveolin-1 Mutation in Mice Drives Inflammatory and Metabolic Defect-Associated Pulmonary Arterial Hypertension
Source: Front Med (Lausanne). 2020 Sep 11;7:540. doi: 10.3389/fmed.2020.00540 (PMC7516012; doi:10.3389/fmed.2020.00540)
Supplement: Supplementary file 1 [file Data_Sheet_1.pdf]

Supplemental Figure Legends

Supplemental Figure 1: Hemodynamic phenotyping of younger wild-type and Cav1 mutant mice. (A) Right ventricular systolic pressure in 8-10-week-old mice. Homozygous mice showed higher pulmonary pressure. **(B)** No significant difference was observed between the groups in the right ventricular hypertrophy (Fulton's Index). **(C)** No difference between the groups in blood glucose level too. Each symbol is the measurement from one animal, with the bar showing mean and SEM.

Supplemental Figure 2: High resolution images of lungs from heterozygous (left) and homozygous (right) Cav1 mutant mice with H&E staining.

Supplemental Figure 3: High-fat diet plus streptozotocin (HFD + STZ) does not alter muscularization in wild-type and Cav1 mutant mice. Muscularized pulmonary vessels, either based on various size **(A)** or coverage **(B)** did not change in Cav1 mutant mice on regular chow or high fat diet plus streptozotocin (HFD + STZ). Numbers are based on the count of vessels in twenty 10x fields in each of four mice per genotype and error bars are standard error of the mean.

Supplemental Figure 4: Cav1 mutant mice trend towards a higher number of $CD68^+$ cells (macrophages or monocytes) in lungs. **(A)** Pictures of $CD68^+$ staining by genotype and diet, **(B)** Quantification, in counts per 20 10x fields, on four mice per condition shows a trend towards more CD68 particularly in Cav1 homozygous mutants, but variability makes it not statistically significant. Each symbol is the measurement from one animal, with the bar showing mean and SEM. **(C)** There were often a higher number $CD68^+$ cells immediately surrounding large muscularized vessels, particularly in Cav1 homozygous mice.
