# Supplementary figures and images for: Expression of a Human Caveolin-1 Mutation in Mice Drives Inflammatory and Metabolic Defect-Associated Pulmonary Arterial Hypertension
Source: Front Med (Lausanne). 2020 Sep 11;7:540. doi: 10.3389/fmed.2020.00540 (PMC7516012; doi:10.3389/fmed.2020.00540)

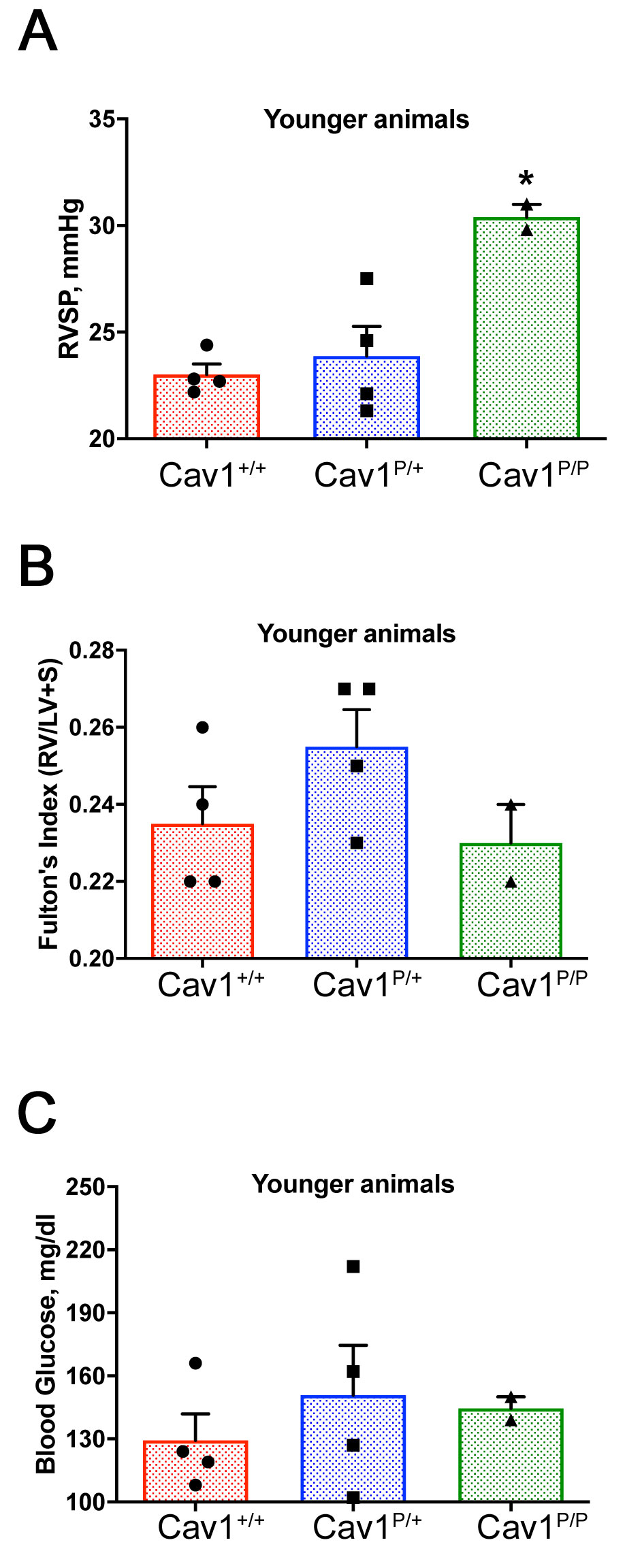

Supplement: Supplementary file 2 [file Image_1.jpg]

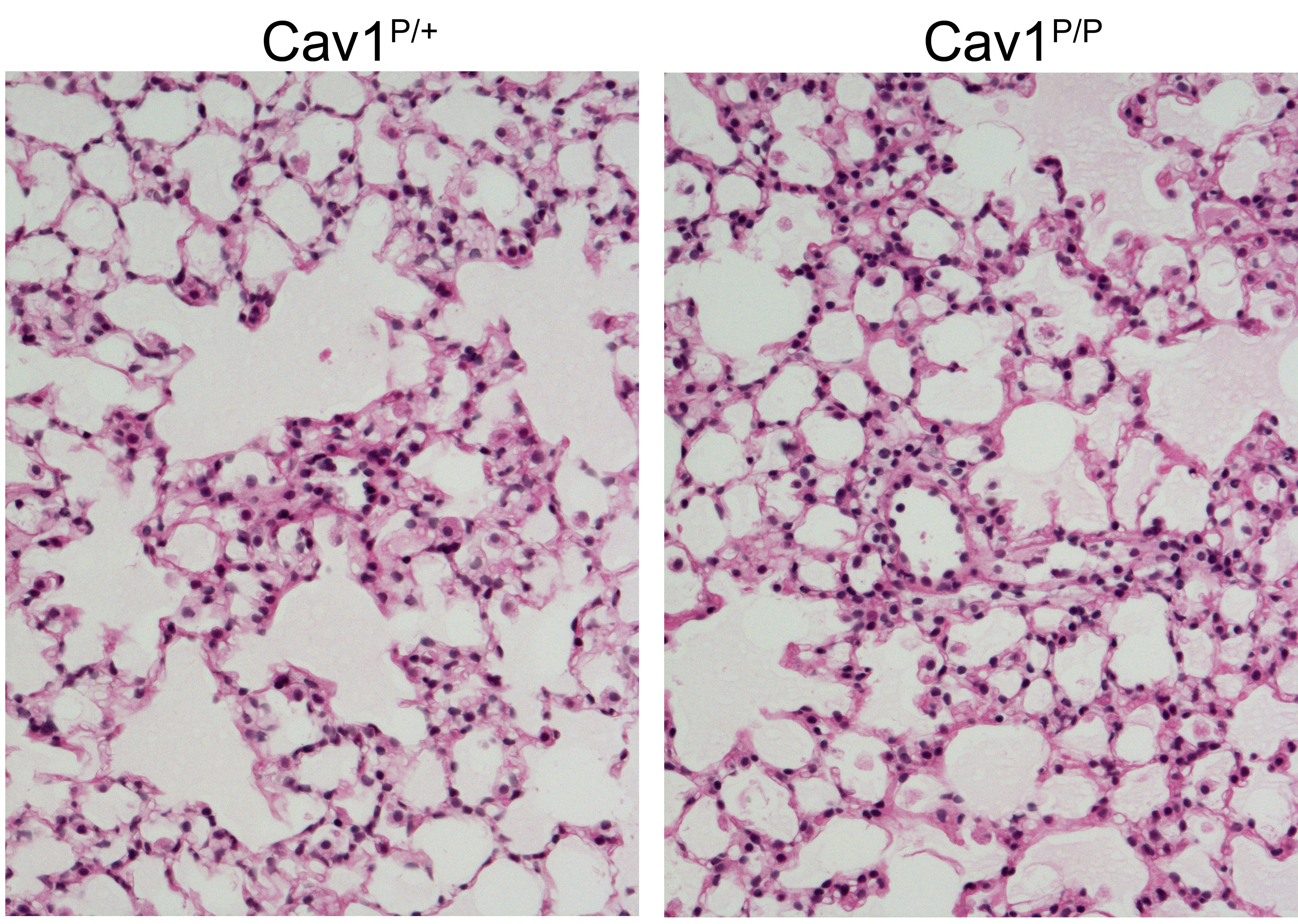

Supplement: Supplementary file 3 [file Image_2.jpg]

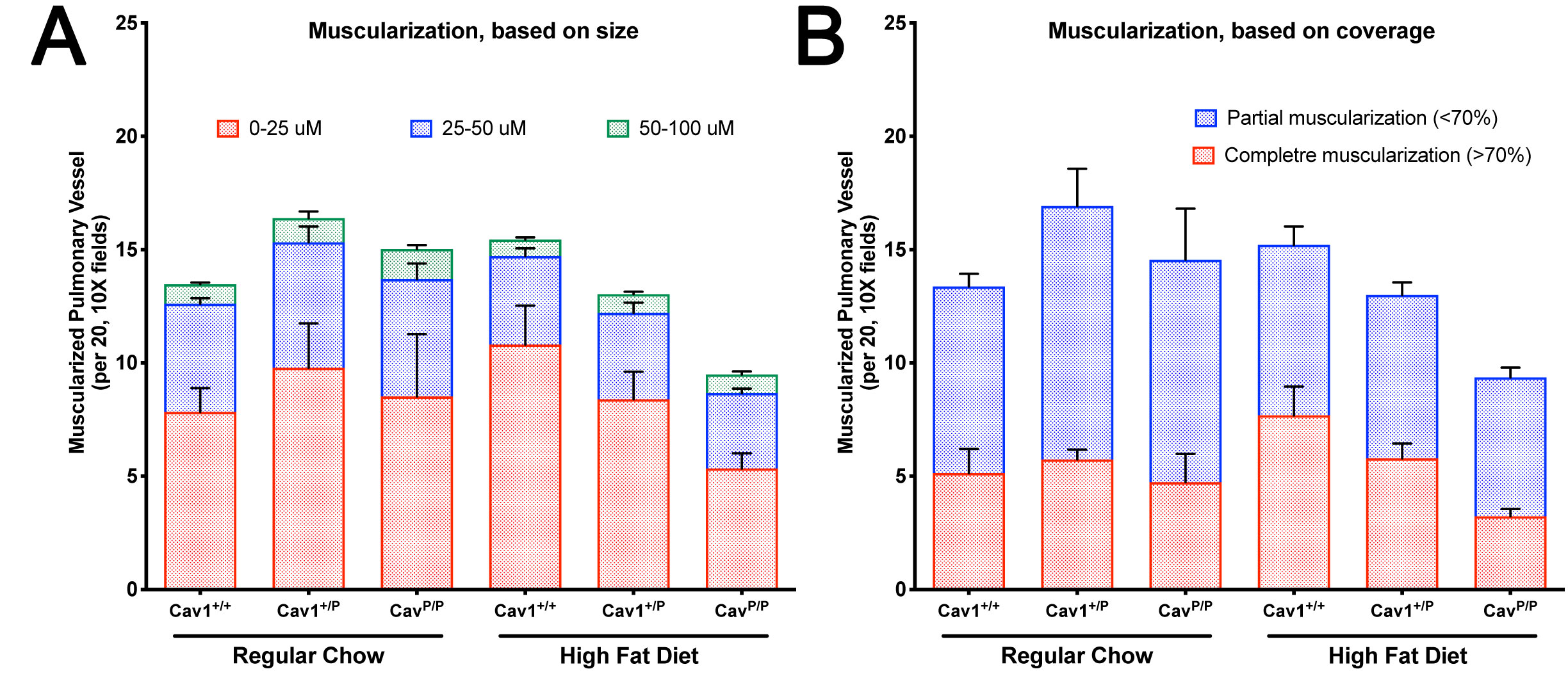

Supplement: Supplementary file 4 [file Image_3.jpg]

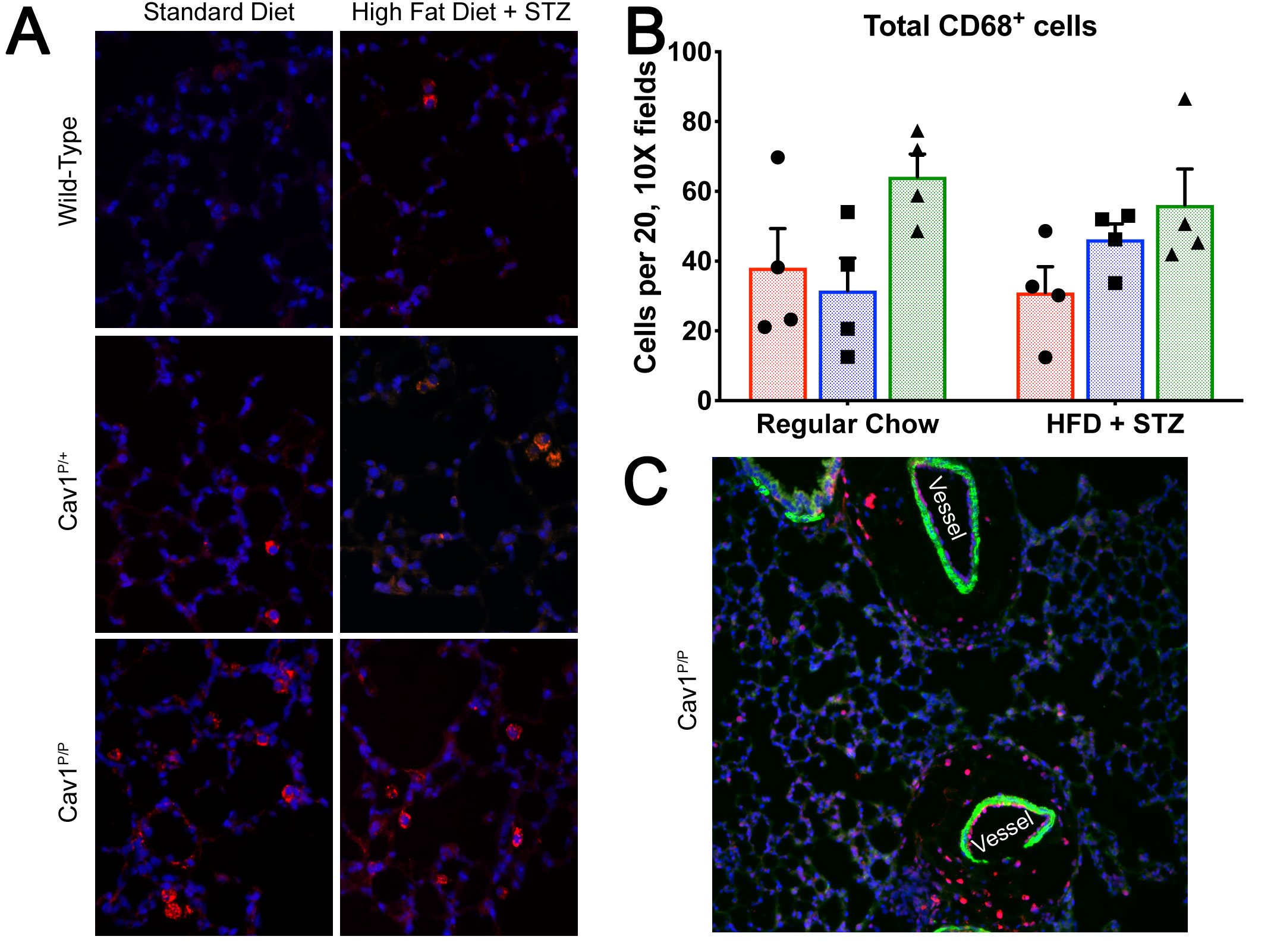

Supplement: Supplementary file 5 [file Image_4.jpg]
